# Anatomical and functional responses in eyes with diabetic macular edema treated with “1 + PRN” ranibizumab: one-year outcomes in population of mainland China

**DOI:** 10.1186/s12886-020-01510-0

**Published:** 2020-06-15

**Authors:** Kunbei Lai, Chuangxin Huang, Longhui Li, Yajun Gong, Fabao Xu, Xiaojing Zhong, Lin Lu, Chenjin Jin

**Affiliations:** State Key Laboratory of Ophthalmology, Zhongshan Ophthalmic Center, Sun Yat-sen University, 54 South Xianlie Road, Guangzhou, 510060 China

**Keywords:** Diabetic macular edema, Ranibizumab, Central foveal thickness, Predictive factor, Pro re nata, Subfoveal choroidal thickness

## Abstract

**Background:**

To evaluate the anatomical and functional responses in eyes with diabetic macular edema (DME) treated with ranibizumab under “1 + pro re nata (PRN)” regimen.

**Methods:**

This prospective interventional case series included 69 eyes of 69 patients with DME treated with intravitreal injections of 0.5 mg ranibizumab followed by repeated injections as needed. Best-corrected visual acuity (BCVA), central foveal thickness (CFT), subfoveal choroidal thickness (SFCT), and predictive factors for final visual outcomes were assessed.

**Results:**

Logarithm of minimal angle of resolution (logMAR) BCVA improved from 0.64 ± 0.23 at baseline to 0.56 ± 0.27, 0.53 ± 0.26, 0.47 ± 0.25, 0.44 ± 0.32, 0.47 ± 0.26 and 0.46 ± 0.26 at time-point of months 1, 2, 3, 6, 9, and 12, respectively (*P* < 0.05 for any follow-up time-point except month 1). CFT decreased from 478.23 ± 172.31 μm at baseline to 349.74 ± 82.21 μm, 313.52 ± 69.62 μm, 292.59 ± 61.07 μm, 284.67 ± 69.85 μm, 268.33 ± 43.03 μm, and 270.39 ± 49.27 μm at above time-points, respectively (*P* < 0.05). The number of injections was 6.83 times over 12 months’ follow-up under “1 + PRN” regimen. Multivariate analysis showed that the factors including age, BCVA at baseline, disruption of ellipsoid zone, posterior vitreous detachment (PVD), and vitreomacular traction (VMT) were correlated with the final BCVA.

**Conclusions:**

Intravitreal injections of ranibizumab under “1 + PRN” regimen is a not only effective but also safe way to improve visual acuity of DME patients. And older age, lower baseline BCVA, VMT, and disruption of ellipsoid zone are predictors for final poor BCVA while PVD is a positive predictive factor for good final BCVA.

**Trial registration:**

The trial was registered retrospectively in ClinicalTrials.gov on 2 June 2019 (NCT03973138).

## Background

Diabetic macular edema (DME) is an important cause of visual impairment in patients with diabetes mellitus (DM), which affects greatly the quality of individual’s life [1–3]. Since the prevalence of diabetes worldwide is increasing, DME has become a global health issue [4, 5]. Laser photocoagulation has once been the standard treatment protocol for DME during the past three decades [6]. Nowadays, treatment of DME shifts to anti-vascular endothelial growth factor (anti-VEGF) therapy [7]. Recent randomized multicenter clinical trials have showed the benefits of anti-VEGF therapy on reducing DME and improving patient’s vision [8–11].

Up to now, most of the studies recommend three loading doses of anti-VEGF injections followed by an as needed/pro re nata (PRN) regimen. However, three loading injections would be a great economic and psychological burden for patients with DME, especially for those who in developing countries. Meanwhile, a proportion of patients actually do not need three loading injections and yet maintain good vision over long period. Therefore, “1 + PRN” regimen, namely one anti-VEGF injection at the very first month, followed by an as needed retreatment protocol might be a treatment option for DME. However, this concept of “1 + PRN” regimen has not been well studied. Herein, we reported the functional as well as anatomical responses in eyes with DME treated with ranibizumab under the “1 + PRN” regimen.

## Methods

### Patients

This study was a prospective, single-center, and interventional study. Prior approval was obtained from the IRB (Institutional Review Board) of Zhongshan Ophthalmic Center of Sun Yat-sen University and all the performance was done in accordance with the tenets of the Declaration of Helsinki. A total of seventy-four patients treated at our hospital (Zhongshan Ophthalmic Center) met the inclusion criteria, however, five patients declined to participate the study for personal reasons, and finally sixty-nine eyes of sixty-nine patients with DME treated at our hospital from January 2015 through June 2019 were enrolled in this study (if both eyes of one patient met the inclusion criteria, the eye with worse VA would enroll in this study). Written informed consents were obtained from all the enrolled patients. The inclusion criteria were: 1) patients aged than 18 years with center-involved DME due to with type 1 or 2 DM who had a best-corrected visual acuity (BCVA) between 20/32 and 20/200, and CFT ≥ 300 μm; 2) DME confirmed by fundus fluorescein angiography (FFA) as well as optical coherence tomography (OCT); 3) decreased vision caused by DME but not any other cause. The exclusion criteria were as follows: 1) previous treatment for DME such as anti-VEGF or laser photocoagulation; 2) patients who required immediate surgery, for example, serious proliferative diabetic retinopathy; 3) other ocular diseases, such as glaucoma, retinal detachment or uveitis; 4) unstable systemic conditions. Our study was registered on https://clinicaltrials.gov (NCT03973138).

### Treatment and follow-up

DME patients were treated by intravitreal injection of ranibizumab (IVR, 0.5 mg; Genentech, USA) at the very first month, followed by a protocol of as needed reinjections until stable vision was achieved over 2 consecutive visits or a BCVA of 20/20 was observed. BCVA, slit-lamp examination, intraocular pressure (IOP) with tonometry, funduscopy, as well as enhanced depth imaging (EDI)-OCT were performed at baseline and monthly routinely. FFA was performed before treatment and when the surgeon considered it was necessary. The primary outcome was the mean change of BCVA at month 12. The secondary outcomes measured the central foveal thickness (CFT), numbers of injections, predictive factors for final BCVA, as well as systemic/ocular adverse events.

### Assessment

Decimal BCVA were transferred to the logarithm of minimal angle of resolution (logMAR) value for analysis. The CFT was determined by the averaging the foveal thickness of the retinal pigment epithelium (RPE) line to inner retinal surface of vertical scan and horizontal scan [12]. Subfoveal choroidal thickness (SFCT) was determined by the vertical distance from the RPE line to the inner surface of choroidal-scleral junction, as previously described [13]. Patients’ characteristics, including the age of onset, gender, duration of DM, duration of DME, BCVA at baseline, CFT, SFCT, continuity of ellipsoid zone (EZ) and external limiting membrane (ELM), as well as other anatomic characteristics of OCT (such as subretinal fluid, intraretinal cysts, posterior vitreous detachment [PVD], and vitreomacular traction [VMT]) at baseline were documented and were analyzed whether they were the predictive factors for one-year visual outcome.

### Statistical analysis

All data was expressed as means ± SD. SPSS 16.0 software (SPSS Corporation) was used for statistical analyses in our study. The *t* test and χ^2^ test were used for continuous variables and categorical variables, respectively. Repeated measure ANOVA was used for the analysis of logMAR VA and CFT. Multivariate analysis was used for the analysis of predictive factors. A *P* < 0.05 (two-tailed) was considered statistically significant for all analysis.

## Results

### Patient characteristics

**Fig. 1:**
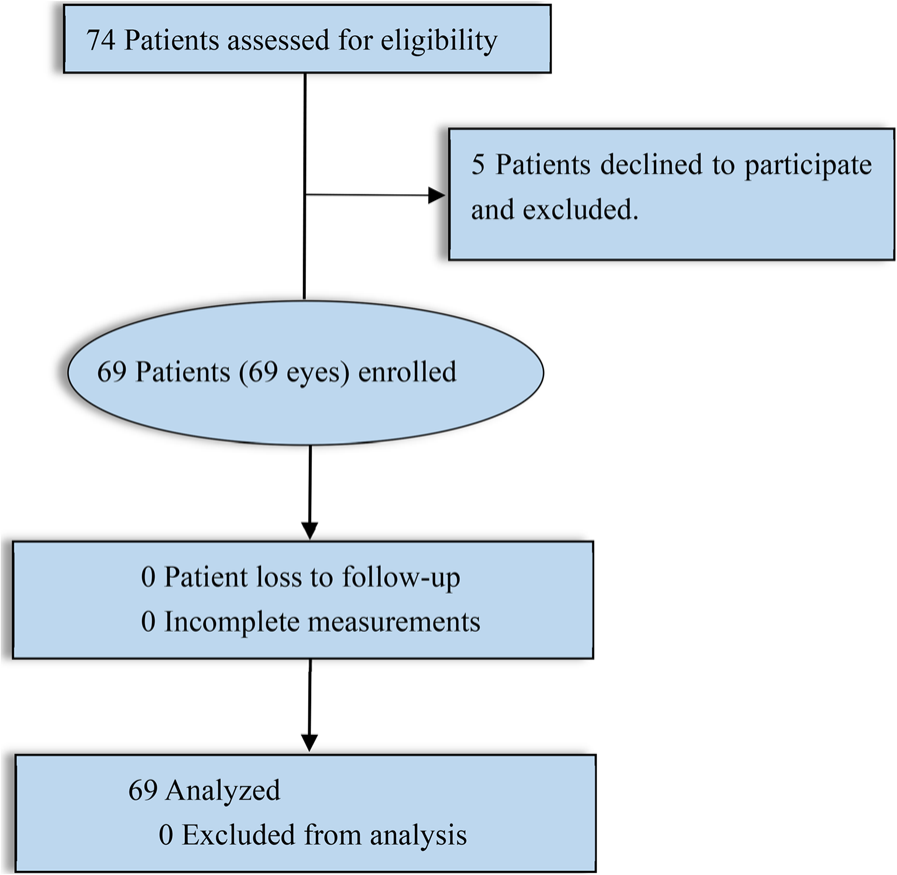
Flowchart for the study

Seventy-four patients who met inclusion criteria, five patients declined to participate the study for personal reasons, and finally sixty-nine eyes of sixty-nine patients enrolled in our study (Fig. 1). Table 1 showed the baseline characteristics of all enrolled cases. There was no statistical difference for the BCVA at baseline between the intact EZ sub-group and the disrupted EZ sub-group (0.61 ± 0.24 vs 0.66 ± 0.22) (*P* > 0.05). Similar results were found for the BCVA at baseline between the intact ELM group and the disrupted ELM group (0.60 ± 0.24 vs 0.68 ± 0.22) (*P* > 0.05). There was no statistical difference for CFT at baseline between subgroups. Thirty-five eyes (50.72%) were mild to moderate non-proliferative diabetic retinopathy (NPDR), twenty-one (30.43%) eyes were severe NPDR, and thirteen eyes (18.84%) were proliferative DR.

**Table 1:** The baseline characteristics of the study patients with diabetic macular edema

Baseline characteristics	*n* = 69
Mean age of onset, mean ± SD	55.75 ± 12.17 (range, 22–78)
Gender	
Male, no. (%)	41 (59.42)
Female, no. (%)	28 (40.58)
BCVA (logMAR units), total, mean ± SD	0.64 ± 0.24
Sub-group of intact EZ	0.61 ± 0.24
Sub-group of disrupted EZ	0.66 ± 0.22
Sub-group of intact ELM	0.60 ± 0.24
Sub-group of disrupted ELM	0.68 ± 0.22
Central foveal thickness (μm), total, mean ± SD	478.23 ± 172.32
Sub-group of intact EZ	476.56 ± 156.02
Sub-group of disrupted EZ	480.40 ± 194.21
Sub-group of intact ELM	481.16 ± 181.04
Sub-group of disrupted ELM	473.38 ± 160.20
Subfoveal choroidal thickness(μm), mean ± SD	229.55 ± 65.07
Percentage of intraretinal fluid cyst, no. (%)	45 (65.22)
Percentage of subretinal fluid, no. (%)	44 (63.77)
Percentage of disrupted EZ, no. (%)	30 (43.48)
Percentage of disrupted ELM, no. (%)	26 (37.68)

*BCVA* best-corrected visual acuity, *logMAR* logarithm of the minimal angle of resolution, *EZ* epiretinal membrane, *ELM* external limiting membrane

### Visual outcome after IVR

**Fig. 2:**
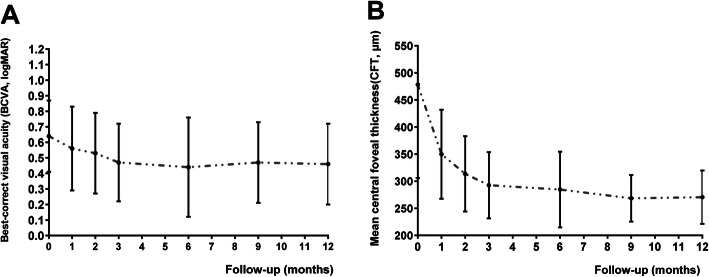
The changes of the mean logarithm of the minimum angle of resolution (logMAR) visual acuity (VA) and central foveal thickness (CFT) during the 12-month follow-up treated with intravitreal injections of ranibizumab (IVR) under “1 + PRN” regimen. **a** showed that logMAR VA decreased with follow-up time-point, there were significant differences for the logMAR VA at each time-point compared with the baseline except month 1 (*P* > 0.05 for month 1, *P* < 0.05 for other follow-up time-points), (**b**) showed that “1 + PRN” IVR treatment significantly reduced the CFT (*P* < 0.05 for all time-points compared with the baseline)

The mean numbers of IVR were 6.83 times during the 12-month follow-up visit. The logMAR VA improved from 0.64 ± 0.23 at the baseline to 0.56 ± 0.27, 0.53 ± 0.26, 0.47 ± 0.25, 0.44 ± 0.32, 0.47 ± 0.26 and 0.46 ± 0.26 at the time-points of months 1, 2, 3, 6, 9, and 12, respectively. Significant differences were found for the logMAR VA at any follow-up compared with that of baseline except the time-point of month 1 (*P* < 0.05 for any follow-up time-point except month 1) (Fig. 2a). Interestingly, for sub-group analysis, the mean BCVA between the intact EZ group and disrupted EZ group was compared, although there was no statically difference at the baseline between the intact EZ group and the disrupted EZ group, we found that the final BCVA was better in the intact EZ group than that of disrupted EZ group (0.61 ± 0.24 vs 0.66 ± 0.22 at the baseline and 0.39 ± 0.24 vs 0.56 ± 0.26 at the final visits) (*P* < 0.01). At month 3, 7 eyes (10.14%) underwent only 1 intravitreal injection, 18 eyes (26.09%) had 2 injections, and the left 44 eyes (63.77%) had 3 injections.

### Central foveal thickness after intravitreal injections of ranibizumab

**Fig. 3:**
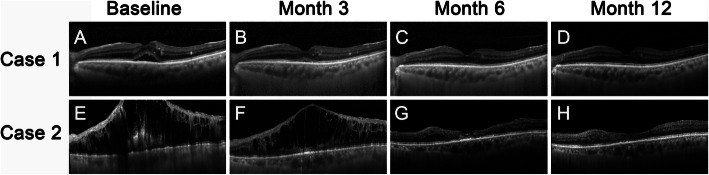
Representative images of patients who received intravitreal injections of ranibizumab (IVR) under “1 + PRN” regimen. **a**-**d** were optical coherence tomography (OCT) images for case 1 at the time-points of baseline, month 3, month 6, and month 12, respectively. Case 1 had a best-corrected visual acuity (BCVA) of 0.3 and intact of external limiting membrane (ELM) and ellipsoid zone (EZ) at baseline (**a**). The subretinal fluid was absorbed immediately after one injection of ranibizumab, and the BCVA increased to 0.6 at month 1. No additional injection was needed for case 1, and the macular remained dry with the BCVA increased to 0.8 at month 12 (**b**-**d**). (E-H) were OCT images for case 2 at baseline, month 3, month 6, and month 12, respectively. Please note that case 2 had a disrupted ELM and EZ with a BCVA of 0.1 at baseline (**e**). Intraretinal fluid was partially absorbed after three injections of ranibizumab (**f**). Case 2 continued to receive another three injections of ranibizumab monthly and then intraretinal fluid was totally absorbed at month 6 (**g**). After a total of 6 times IVR under “1 + PRN” regimen the intraretinal fluid was totally absorbed, however, the BCVA was still 0.1 at month 12

Mean ± SD central foveal thickness was 478.23 ± 172.31 μm at baseline, and it decreased to 349.74 ± 82.21 μm, 313.52 ± 69.62 μm, 292.59 ± 61.07 μm, 284.67 ± 69.85 μm, 268.33 ± 43.03 μm, and 270.39 ± 49.27 μm at above follow-up time-points, respectively (*P* < 0.05). But there was no difference neither between the intact EZ sub-group and the disrupted EZ sub-group nor between the intact ELM sub-group and the disrupted ELM sub-group at any time-point (*P* > 0.05) (Fig. 2b). Representative images are shown in Fig. 3.

### Subfoveal choroidal thickness after IVR

SFCT at baseline and one-year after IVR were compared in this study, although there was statistical difference between the SFCT at baseline and SFCT at month 12 after IVR under “1 + PRN” regimen (229.55 ± 65.07 μm vs 209.91 ± 63.74 μm, *P* < 0.05), multivariate linear regression analysis demonstrated that SFCT at baseline was not a predictive factor for BCVA at one-year follow-up (*P* > 0.05).

### Predictive factors for one-year visual prognosis

Multivariate linear regression analysis demonstrated that the predictors for final VA were the age (*P* = 0.013), presence of VMT (*P* = 0.005), BCVA at baseline (*P* = 0.001), PVD development (*P* = 0.004), and EZ disruption (*P* = 0.01) (Table 2). Older age, poor baseline BCVA, presence of VMT, as well as EZ disruption were more at risk of poor final VA than eyes without these findings, while development of PVD was associated with good final VA.

**Table 2:** Multivariate analysis of predictive factors for final best corrected visual acuity

Variables	Unstandardized coefficients		Standardized coefficients	*P*	95% confidence interval for B	
	B	SE	β		Lower bound	Upper bound
Age	−0.005	0.002	−0.233	0.013^a^	−0.009	− 0.001
Gender	0.086	0.043	0.161	0.052	0.000	0.172
DM duration	0.011	0.007	0.135	0.155	−0.004	0.026
DME duration	−0.027	0.035	−0.068	0.438	−0.096	0.042
BCVA at baseline	0.498	0.096	0.441	0.001^a^	0.305	0.691
CFT	0.000	0.000	0.000	0.994	0.000	0.000
Choroidal thickness	0.000	0.000	−0.046	0.586	0.000	0.000
Intraretinal cysts	0.001	0.046	0.002	0.980	−0.092	0.094
SRF	0.034	0.047	0.066	0.471	−0.060	0.129
PVD	−0.144	0.048	−0.275	0.004^a^	−0.241	−0.047
VMT	0.231	0.078	0.284	0.005^a^	0.075	0.388
ERM	0.087	0.078	0.101	0.269	−0.069	0.243
Disrupted EZ	0.155	0.058	0.294	0.010^a^	−0.006	0.235
Disrupted ELM	0.104	0.064	0.193	0.108	−0.024	0.231
Disrupted RPE layer	−0.033	0.05	−0.064	0.506	−0.133	0.067

*SE* standard error, *DM* diabetes mellitus, *DME* diabetic macular edema, *BCVA* best corrected visual acuity, *CFT* central foveal thickness, *SRF* subretinal fluid, *PVD* posterior vitreous detachment, *VTM* vitreomacular traction, *ERM* epiretinal membrane, *EZ* ellipsoid zone, *ELM* external limiting membrane, *RPE* retinal pigment epithelium

^a^Statistically significant result

### Ocular/systemic complications

No systemic complication was found in the study. Although there were three patients who experienced transient IOP elevation which became normal on the second day, other ocular complication was not detected in any of the patients.

## Discussion

In this present study, our data showed that intravitreal injections of ranibizumab under “1 + PRN” regimen led to significant improvements in BCVA and reduction of the CFT over 12 months. Besides, our data revealed that older age, poor baseline BCVA, presence of VMT, as well as EZ disruption were more at risk of poor final VA than eyes without these findings, while development of PVD was associated with good final VA at month 12.

VEGF is an important mediator which responses for the abnormal vascular permeability in DME [14, 15]. Ranibizumab, a recombinant humanized monoclonal antibody for VEGF-A [16, 17], was approved by FDA for indication of DME in 2012. Many clinical trials, including READ-2 study [18, 19], the RESOLVE study [9, 20], the Diabetic Retinopathy Clinical Research Network (DRCR.net) study [7, 21, 22], the RESTORE study [9], the REVEAL study [23], RISE and RIDE study [24], and the REFINE study [11], have demonstrated the safety and effectiveness of IVR for treating DME. In some previous clinical trials, continuous monthly injections of ranibizumab has been recommended, which may optimize the efficacy of treatment [25]. However, monthly injections are not practical in the real world, therefore, ophthalmologists are now seeking other alternative treatment regimens. Three loading doses of anti-VEGF injections followed by a PRN protocol is now widely adopted by clinicians for treating center-involved DME [11, 25, 26]. However, three loading injections could still be a great economic and psychological burden for patients with DME, especially for those who live in developing countries. Meanwhile, a proportion of patients actually do not need the three loading injections and yet maintain good vision over long period [27], therefore, for these patients three loading doses may be unnecessary and would be a waste of money. Recently, ophthalmologists are focus on other treatment modalities like the treat-and-extend regimen [28] and “1 + PRN” regimen for the management of DME.

There are still very few studies evaluating the effectiveness of “1 + PRN” regimen for treating center-involved DME. In our study, we demonstrated that more than one third of the cases did not need three loading doses at month 3, which was consistent with the findings showed by James et al. [26]. James reported that 24 out of 180 eyes (13%) need only one injection of ranibizumab, 52 out of 180 eyes underwent 2 injections, and the left 104 eyes had 3 injections on a monthly basis [26]. Therefore, James considered that there would be considerable reduction in health care cost if one third of patients did not received the three loading doses for treating DME [26]. Further, in a recent retrospective study, Ebneter et al. compared outcomes of PRN injections based on BCVA versus OCT-based treat-and-extend regimen for DME, and they found that two groups had similar visual acuity outcomes, but patients in the PRN injections group based on BCVA received significantly fewer intravitreal injections than patients in the OCT-based treat-and-extend regimen group [28]. Specifically, the mean number of the IVR was 6.83 times during the one-year follow-up visit in our study, which was similar with the mean number of 5 times reported by James et al. under “1 + PRN” regimen [26]. Interestingly, this mean number of 6.83 injections under “1 + PRN” regimen in our study was relatively fewer than the number of 7.9 injections reported by REFINE study [11] and average 7 injections reported by RESTORE study under “3 + PRN” regimen [9]. In our study, the logMAR VA improved from 0.64 before treatment to 0.46 at the final visit of month 12, namely increasement of 0.18 logMAR VA, which was similar with “3 + PRN” studies such as REFINE study [11] and RESTORE study [9] but lower than the increasement of 0.29 logMAR VA under monthly injection regimen reported by Nepomuceno et al. [29]. In addition, in our study, the mean CFT decreased from 478.23 μm before treatment to 270.39 μm at final visit of month 12, which was a reduction of 207.84 μm of CFT for the patients over one-year “1 + PRN” treatment. Interestingly, the mean changes of CFT varied in different studies with different treatment regimens. The REFINE study showed a mean reduction of 146.5 μm for CFT after 12 months’ treatment under “3 + PRN” regimen [11]. The RISE and RIDE study reported a mean reduction of 249.3 μm for CFT after monthly intravitreal injections of ranibizumab for 12 months [30] while Nepomuceno et al. reported a mean reduction of 126 μm for CFT under monthly injection regimen [29]. We believed that the mean changes of BCVA and CFT varied not only related to the treatment regimens but also related to other factors such as inclusion criteria and exclusion criteria. Therefore, future randomized controlled clinical trials are still needed to compared the efficacy between “1 + PRN” and other treatment regimens.

It is important for the ophthalmologists to know whether baseline characteristics would predict the final visual outcome for the patients treated by the ranibizumab. Sophie et al. found that good baseline BCVA was a predict factor for final BCVA of 20/40 or better, and submacular fluid and severe cystic edema were predict factors for poor visual outcome without treatment but respond well when treated with monthly IVR [31]. Channa et al. revealed that poor baseline BCVA predicted poor visual outcomes [32]. Yucel et al. reported that older age, female, poor BCVA at baseline, VMT, and disruption of EZ were predictors for final poor BCVA prognosis, while PVD and leaking microaneurysms were predictors for the good final BCVA [27]. Our “1 + PRN” regimen data had similar results with previous studies [27, 31–33]. Interestingly, in our study we found it that although there was statically difference between the SFCT at baseline and SFCT at 12 months’ follow-up time-point after intravitreal injections of ranibizumab under “1 + PRN” regimen, multivariate linear regression analysis demonstrated that SFCT at baseline was not a predictive factor for BCVA at the final visit of month 12. Actually, there is still controversy on the role of SFCT for predicting the VA outcome. Rayess et al. reported that greater baseline SFCT were associated with better anatomic and functional responses [34]. Similarly, Nourinia et al. reported in their prospective interventional case series that SFCT reduction was correlated with CFT reduction as well as vision improvement [35]. However, Campos et al. found that baseline SFCT decreased was not a predictor for anatomic or functional outcome for treatment of DME outcome [36]. In our study, although there was statically difference between the SFCT at baseline and SFCT at month 12 after intravitreal injections of ranibizumab under “1 + PRN” regimen, multivariate linear regression analysis demonstrated that SFCT at baseline was not a predictive factor for BCVA at the final visit of month 12. However, the role of SFCT still requires further investigations.

Our study has some limitations including relatively small number of patients and short-term follow-up visit. Besides, although we have full data of the patients treated with ranibizumab, there was no control group in our study. Even with these limitations, our results have enough strength to conclude that the intravitreal injections of ranibizumab under “1 + PRN” regimen is a safety and effective way to improve BCVA and reduce the CFT of DME patients, which could be an option for the treatment of center-involved DME.

## Conclusion

In summary, based on our results, intravitreal injections of ranibizumab under “1 + PRN” regimen could significantly improve BCVA and reduce the CFT and SCFT over 12 months. Older age, poor baseline BCVA, presence of VMT, and EZ disruption were more at risk of poor final VA, while development of PVD was associated with final good BCVA at one-year follow-up visit.

## Data Availability

The datasets used and/or analysed during the current study are available from the corresponding author on reasonable request.

## Abbreviations

DME: Diabetic macular edema
VEGF: Vascular endothelial growth factor
PRN: Pro re nata
BCVA: Best-corrected visual acuity
CFT: Central foveal thickness
FFA: Fundus fluorescein angiography
OCT: Optical coherence tomography
EDI: Enhanced depth imaging
logMAR: Logarithm of minimal angle of resolution
RPE: Retinal pigment epithelium
SFCT: Subfoveal choroidal thickness
EZ: Ellipsoid zone
ELM: External limiting membrane
PDR: Proliferative diabetic retinopathy
SRF: Subretinal fluid
PVD: Posterior vitreous detachment
VMT: Vitreomacular traction.
